# Efficacy of Epinastine Eyelid Cream in Pediatric Vernal Keratoconjunctivitis: A Case Report

**DOI:** 10.7759/cureus.74268

**Published:** 2024-11-22

**Authors:** Noriyasu Hashida, Rina Hirose, Daiki Shiozaki, Yukinori Mamoto

**Affiliations:** 1 Ophthalmology, Osaka University, Suita, JPN; 2 Ophthalmology, Nakanoshima Eye Center Clinic, Osaka, JPN; 3 Ophthalmology, Mamoto Eye Clinic, Higashi-Osaka, JPN

**Keywords:** allergic conjunctivitis, epinastine, eyelid cream, treatment, vernal keratoconjunctivitis

## Abstract

Allergic conjunctivitis (AC) is characterized by inflammatory responses in the conjunctiva and is often complicated by atopic dermatitis and mechanical irritation. Vernal keratoconjunctivitis (VKC), a severe subtype of AC, presents unique challenges in its diagnosis and management, particularly in pediatric patients. This case report describes an eight-year-old girl with VKC who exhibited poor adherence to a prescribed regimen of 0.05% epinastine ophthalmic solution and corticosteroid eye drops, resulting in persistent symptoms. Despite initial treatment, the patient’s condition included giant papillae and thickened tarsal conjunctivae that were resistant to standard therapies. Upon transitioning to 0.5% epinastine eyelid cream, the patient showed improved adherence and partial symptom resolution. However, new limbal lesions, including Horner-Trantas dots, were observed following the cessation of steroid therapy. The reintroduction of steroids led to significant improvements in conjunctival lesions and complete resolution of limbal gelatinous hyperplasia. This case underscores the potential of epinastine eyelid cream in managing VKC, particularly in patients with adherence issues, and highlights the need for careful monitoring and combination therapy in severe cases. Further studies are needed to evaluate the long-term efficacy and safety of epinastine eyelid cream. However, once-daily application of eyelid cream may serve as a viable treatment and management option for pediatric patients with VKC.

## Introduction

Allergic conjunctivitis (AC) is an inflammatory disease of the conjunctiva initially mediated by antigen exposure and subsequent immune response of group 2 innate lymphoid cell (ILC-2), type 2 helper T (Th2) lymphocyte activation, and involvement of cytokines and chemokines [1-4]. Based on the presence of proliferative changes in the conjunctiva, complications of atopic dermatitis, and mechanical irritation by foreign substances, they are classified into several types including atopic keratoconjunctivitis (AKC), vernal keratoconjunctivitis (VKC), and giant papillary conjunctivitis (GPC). VKC, a subtype of AC, is characterized by hypertrophic papillae with proliferative changes in the conjunctiva, leading to a cobblestone appearance of the upper tarsal conjunctiva, Horner-Trantas dots in the limbic area, and various corneal complications such as corneal shield ulcers [1,2,5]. Diagnosis and management of VKC, especially in children, are challenging because the variety of clinical manifestations and difficulties in drug management contribute to the complexity of the disease and result in chronic conditions [6,7].

The treatment of AC primarily involves the use of chemical mediator release inhibitors, histamine receptor antagonists, cyclosporine A, and tacrolimus as immunosuppressive agents [8,9]. In particular, as a first-line treatment for VKC, immunosuppressant eye drops are used in conjunction with anti-allergic eye drops [1,2,9]. In severe cases, combination therapy comprising immunosuppressive eye drops, anti-allergic eye drops, and a topical steroid is administered. The alleviation of itching and inflammation through the use of eye drops is crucial in managing AC. However, the potential for treatment failure due to lack of adherence increases with the frequency of eye drop administration.

Epinastine hydrochloride, a histamine receptor antagonist and mast cell stabilizer [10,11], is an antihistamine commonly used to treat AC [12-14]. Most of its applications are limited to eye drops, such as 0.05% and 0.1% epinastine ophthalmic solution, with respective instillation frequencies of four times/day and two times/day is required. A new product has recently been developed, 0.5% epinastine eyelid cream, which has been demonstrated to be highly effective when applied once to the eyelid skin [15,16]. Lower doses may result in improved adherence to the management of long-term allergic symptoms. The efficacy of epinastine eyelid cream in the treatment of AC has been well documented [15,16]; however, there is a paucity of evidence regarding its efficacy in the more severe form of VKC, which is associated with proliferative changes in the eyelid conjunctiva.

In the current study, we report a case of AC and VKC in which the patient exhibited poor adherence and uncontrolled itching. The application of epinastine eyelid cream led to an improvement in adherence and a mild reduction in conjunctival lesions.

## Case presentation

The patient was an eight-year-old girl. She experienced itchy eyes for six months and visited a local ophthalmologist who diagnosed her with VKC based on the observation of giant cobblestone papillae, thickened tarsal conjunctivae, and mucous discharge of the conjunctiva. The initial treatment regimen involved the administration of 0.05% epinastine ophthalmic solution four times daily. However, the patient did not adhere to the prescribed frequency, which resulted in persistent itching and redness. To better control symptoms, 0.1% fluorometholone and 0.5% levofloxacin hydrate eye drops were added to the treatment plan. The patient was referred to our hospital for the management of ocular itching and allergic reactions because the itching had subsided with the use of epinastine and steroid eye drops although symptoms had not completely disappeared.

**Figure 1:**
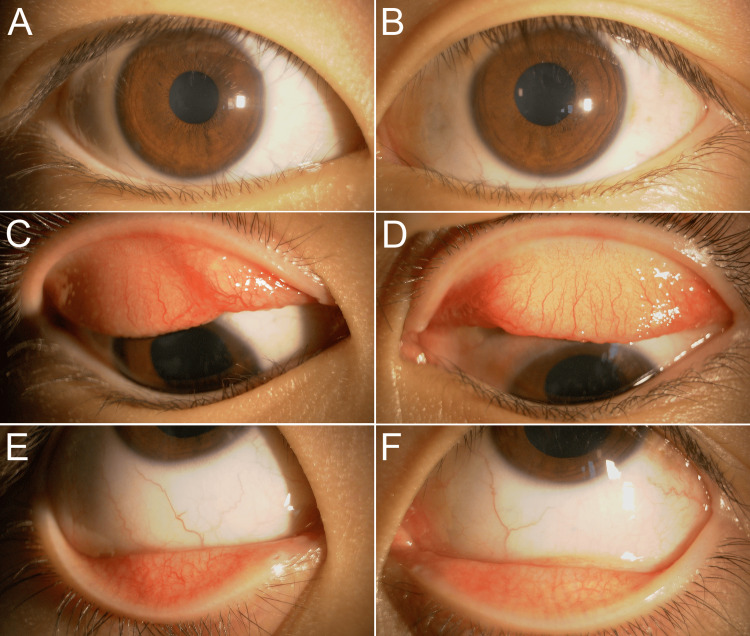
Anterior segment findings at the initial examination (A, B) Mild conjunctival hyperemia with no corneal abnormalities. (C, D) Congestion and swelling of the palpebral conjunctiva with giant papillae. (E, F) Conjunctival hyperemia of the lower eyelid.

The best-corrected visual acuity (BCVA) was 20/20 in both eyes, and intraocular pressure was normal. Giant papillae and thickened tarsal conjunctivae, coupled with hyperemia and limbal vascularization, were indicative of chronic VKC (Figures 1A-1D). Based on detailed dermatological evaluation and thorough parental interviews, no documented history of atopic dermatitis was found. The patient had no history of pet ownership. Laboratory analysis demonstrated that she exhibited an allergic response to cedar and cypress pollen. No other hematological abnormalities were observed. On initial examination, minimal bulbar conjunctival injection was performed in both eyes (Figures 1A, 1B). Although regression was noted in the giant papillae, persistent bilateral conjunctival hyperemia of the palpebral conjunctiva indicated the persistence of allergic symptoms (Figures 1C, 1D, 3A, 3B). Corneal fluorescein staining was unremarkable in both eyes. In addition, mild hyperemia of the lower palpebral conjunctiva was observed (Figures 1E, 1F).

**Figure 2:**
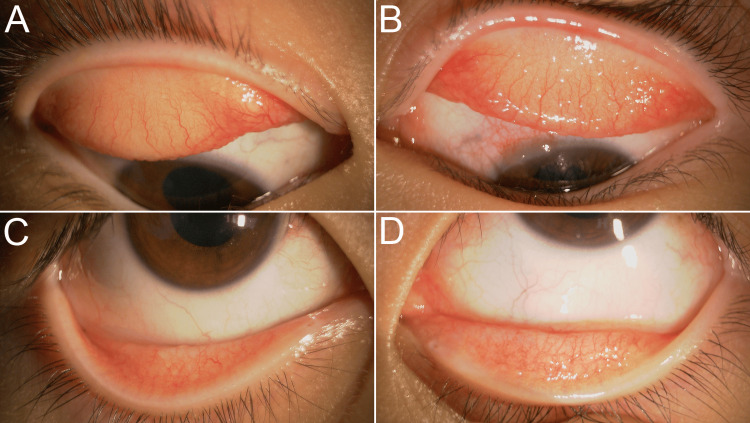
Conjunctival finding one month after the epinastine eyelid cream In the right eye, the patient no longer experiences itching, and improvement in the giant papillae of the upper eyelid is observed (A, B). However, in the left eye, giant papillae increase with worsening conjunctival hyperemia (C, D). Limbal gelatinous infiltrates are observed in the left eye (B).

**Figure 3:**
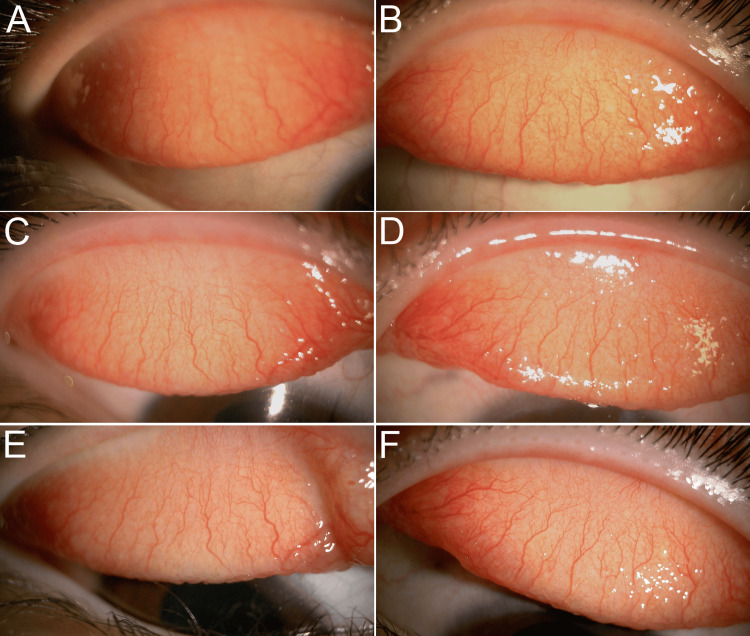
Progression of the upper eyelid (A, B) Initial presentation showed congestion and swelling of the palpebral conjunctiva with giant papillae. (C, D) One month after starting epinastine eyelid cream, improvement in the giant papillae of the upper eyelid in the right eye was observed, while giant papillae enlargement and increased conjunctival hyperemia were noted in the left eye. (E, F) Following initiation of topical steroid therapy, remission was maintained at one-month post-treatment.

**Figure 4:**
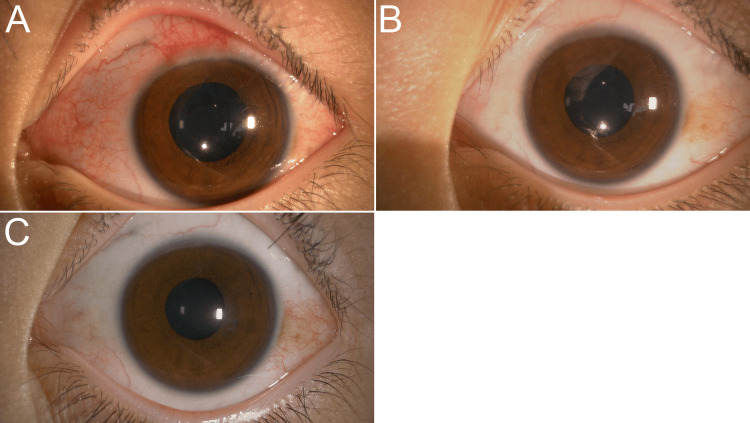
Progression of limbal findings One month after the patient discontinues steroid eye drops, a limbal examination reveals Horner-Trantas dots and conjunctival hyperemia (A). Two weeks after restarting steroids, these findings improve (B), and remission is maintained one-month post-treatment (C).

Epinastine eye drops were replaced with epinastine eyelid cream. One month after initiating this therapy, the giant papillae and conjunctival hyperemia in the right eye improved (Figures 2A, 2C, 3C). However, new lesions, including Horner-Trantas dots and conjunctival papillary changes, emerged in the limbus of the left eye (Figures 2B, 2D, 3D, 4A).

Further investigation revealed that the patient discontinued the prescribed steroid eye drops. Steroid therapy was reintroduced, and within two weeks, the bilateral palpebral conjunctival lesions improved remarkably (Figures 3E, 3F), with complete resolution of the Horner-Trantas dots (Figures 4B, 4C). Steroid therapy was discontinued after two weeks upon achieving symptomatic improvement.

**Figure 5:**
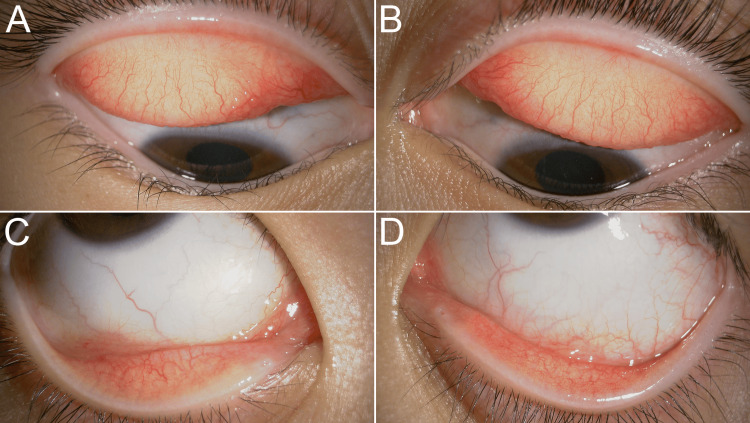
Palpebral conjunctival findings three months after treatment With the use of epinastine eyelid cream and fluorometholone eye drops, the patient experienced complete relief from itching, along with improvement in conjunctival proliferative changes (A, B) and a reduction in hyperemia (C, D).

Epinastine eyelid cream was applied consistently and without interruption throughout the entire treatment period. Three months after starting the administration of epinastine eyelid cream, AC and VKC were well managed without any adverse drug reactions (Figures 5A-5D). Currently, the patient is being followed up without complications. The subsequent clinical course was uneventful.

## Discussion

VKC represents a particularly challenging subtype of AC, primarily because of its complex clinical manifestations and difficulty in managing its symptoms, especially in pediatric patients [1,2,5-7]. Immunosuppressive agents and histamine receptor antagonists are essential components of the standard treatment regimen for VKC [1,2,8,9]. During disease control periods, maintenance therapy with anti-allergic eye drops is frequently employed. However, in contrast to the acute phase, management of the chronic phase of VKC presents a significant challenge, particularly regarding treatment adherence. In the current case, we successfully improved adherence and managed allergic symptoms, including pruritus, by prescribing epinephrine eyelid cream to a pediatric patient with VKC who had poor adherence owing to the frequent use of eye drops. Failure to maintain the consistent use of these medications likely contributes to the persistence of symptoms, underscoring the importance of developing treatment protocols that enhance patient compliance, particularly in younger populations. Although the efficacy of epinastine eyelid cream for SAC has been well-documented [17], there have been no reports addressing its effectiveness for VKC, a condition not represented in clinical trial entries. This case report presents observations on the management of the chronic phase VKC with epinastine eyelid cream, highlighting its potential utility in this context.

The introduction of epinastine eyelid cream as an alternative to traditional eye drops improved adherence, leading to a reduction in conjunctival lesions in the right eye. However, the emergence of new lesions in the left eye, including limbal gelatinous hyperplasia, following the cessation of steroid therapy raises important considerations regarding the role of combination therapy in managing VKC. This case suggests that while epinastine eyelid cream may be effective in improving compliance and managing allergic symptoms, it may not be sufficient as a monotherapy in severe cases where a more aggressive approach, including the use of steroids, may be necessary. The resolution of Horner-Trantas dots and the overall improvement following the reintroduction of steroid therapy highlight the critical role of steroids in controlling severe VKC symptoms [5,8]. This case emphasizes the need for ongoing monitoring and a tailored treatment approach that balances the benefits of improved adherence with the need to control inflammation through the appropriate use of steroids.

Chronic conjunctivitis requires a year-round treatment with various eye drops. In particular, antihistamine eye drops play an important role in the treatment and management of AC [13]. While epinastine eyelid cream is highly effective in controlling itching, it cannot suppress inflammatory mediators such as cytokines and chemokines, which are critical in the development of proliferative lesions. Epithelial cell-derived, interleukin (IL)-33, and thymic stromal lymphopoietin (TSLP), along with ILC-2, have become widely recognized as pivotal players in the pathogenesis of allergic inflammation [8]. The signaling pathways mediated by these factors result in the production of IL-5 and IL-13 by ILC-2, while Th2 lymphocytes secrete IL-4, IL-13, and IL-31. Subsequently, the actions of IL-4 and IL-13 drive the production of IgE, whereas IL-31 induces pruritus [2-4,8]. Epinastine eyelid cream inhibits histamine-mediated pathways but cannot serve as a fundamental treatment due to its inability to suppress cytokine release. Moreover, it does not inhibit IL-31-mediated pathways, such as those responsible for eyelid pruritus associated with atopic dermatitis. Therefore, in severe cases, steroid therapy targeting these factors is necessary. These observations suggest that epinastine cream alone may not be adequate for managing the severe proliferative aspects of VKC. The production of histamine and other pruritic chemical mediators is primarily driven by activated mast cells and basophils [10,11]. Additionally, the inflammatory response mediated by cytokines and chemokines involves tissue mast cells, eosinophils, and Th2 lymphocytes [2,18,19]. Epinastine effectively suppresses eosinophil-associated inflammation by inhibiting eosinophil survival and effectively reducing giant papillae [20]. However, it did not significantly inhibit other inflammatory cells or the production of inflammatory mediators, which may explain its limited effectiveness in preventing the development of limbal lesions.

The management of chronic-phase VKC necessitates a meticulous assessment of inflammatory activity at each visit, guided by three principal clinical parameters: subjective symptoms, the degree of conjunctival hyperemia, and the presence or absence of conjunctival edema. A therapeutic regimen comprising baseline administration of epinastine eyelid cream, augmented with immunosuppressive or corticosteroid eye drops during exacerbations, represents an optimal approach. Strict adherence to once-daily application is paramount during the initial phase. However, pharmacokinetic analyses reveal that effective drug concentrations within the eyelid are sustained for up to 72 hours [16], supporting the feasibility of a tapered regimen with intermittent applications every few days during the stable phase of VKC.

## Conclusions

Further studies are needed to establish the long-term efficacy and safety of epinastine eyelid cream, particularly in combination with other therapies, for the management of VKC. However, the eyelid cream alleviated itching breaking the cycle of the allergic process and reducing proliferative changes in the conjunctiva.

## References

[1] BenEzra D, Pe'er J, Brodsky M, Cohen E (1986). Cyclosporine eyedrops for the treatment of severe vernal keratoconjunctivitis. Am J Ophthalmol.

[2] Leonardi A (2002). Vernal keratoconjunctivitis: pathogenesis and treatment. Prog Retin Eye Res.

[3] Uchio E, Ono SY, Ikezawa Z, Ohno S (2000). Tear levels of interferon-gamma, interleukin (IL) -2, IL-4 and IL-5 in patients with vernal keratoconjunctivitis, atopic keratoconjunctivitis and allergic conjunctivitis. Clin Exp Allergy.

[4] Zheng H, Zhang Y, Pan J (2021). The role of type 2 innate lymphoid cells in allergic diseases. Front Immunol.

[5] Bruschi G, Ghiglioni DG, Cozzi L, Osnaghi S, Viola F, Marchisio P (2023). Vernal keratoconjunctivitis: a systematic review. Clin Rev Allergy Immunol.

[6] Mahoney MJ, Bekibele R, Notermann SL, Reuter TG, Borman-Shoap EC (2023). Pediatric conjunctivitis: a review of clinical manifestations, diagnosis, and management. Children (Basel).

[7] Berger WE, Granet DB, Kabat AG (2017). Diagnosis and management of allergic conjunctivitis in pediatric patients. Allergy Asthma Proc.

[8] Leonardi A, Quintieri L, Presa IJ (2024). Allergic conjunctivitis management: update on ophthalmic solutions. Curr Allergy Asthma Rep.

[9] Shoji J, Ohashi Y, Fukushima A (2019). Topical tacrolimus for chronic allergic conjunctival disease with and without atopic dermatitis. Curr Eye Res.

[10] Kamei C, Akagi M, Mio M, Kitazumi K, Izushi K, Masaki S, Tasaka K (1992). Antiallergic effect of epinastine (WAL 801 CL) on immediate hypersensitivity reactions: (I). Elucidation of the mechanism for histamine release inhibition. Immunopharmacol Immunotoxicol.

[11] Kamei C, Mio M, Kitazumi K, Tsujimoto S, Yoshida T, Adachi Y, Tasaka K (1992). Antiallergic effect of epinastine (WAL 801 CL) on immediate hypersensitivity reactions: (II). Antagonistic effect of epinastine on chemical mediators, mainly antihistaminic and anti-PAF effects. Immunopharmacol Immunotoxicol.

[12] Tasaka K (2000). Epinastine: An update of its pharmacology, metabolism, clinical efficacy and tolerability in the treatment of allergic diseases. Drugs Today (Barc).

[13] Whitcup SM, Bradford R, Lue J, Schiffman RM, Abelson MB (2004). Efficacy and tolerability of ophthalmic epinastine: a randomized, double-masked, parallel-group, active- and vehicle-controlled environmental trial in patients with seasonal allergic conjunctivitis. Clin Ther.

[14] Friedlaender MH (2006). Epinastine in the management of ocular allergic disease. Int Ophthalmol Clin.

[15] Ogura N, Fujisawa K, Kato M (2024). Epinastine cream: a novel once-daily therapeutic agent for allergic conjunctivitis. J Ocul Pharmacol Ther.

[16] Mochizuki T, Hata T, Mori N, Yamazaki T, Noto T, Mano H (2024). Trans-eyelid distribution of epinastine to the conjunctiva following eyelid application in rabbits. Jpn J Ophthalmol.

[17] Fujishima H, Shoji J (2024). Safety and efficacy of a novel 0.5% epinastine topical eyelid cream in allergic conjunctivitis: a phase 3 trial [PREPRINT]. Jpn J Ophthalmol.

[18] Hingorani M, Calder V, Jolly G, Buckley RJ, Lightman SL (1998). Eosinophil surface antigen expression and cytokine production vary in different ocular allergic diseases. J Allergy Clin Immunol.

[19] Abu El-Asrar AM, Struyf S, Al-Kharashi SA, Missotten L, Van Damme J, Geboes K (2000). Chemokines in the limbal form of vernal keratoconjunctivitis. Br J Ophthalmol.

[20] Watase F, Watanabe S, Kanai K (2008). Modulation of eosinophil survival by epinastine hydrochloride, an H1 receptor antagonist, in vitro. In Vivo.

